# Thinking in circuits: toward neurobiological explanation in cognitive neuroscience

**DOI:** 10.1007/s00422-014-0603-9

**Published:** 2014-06-18

**Authors:** Friedemann Pulvermüller, Max Garagnani, Thomas Wennekers

**Affiliations:** 1 Brain Language Laboratory, Department of Philosophy and Humanities, Cluster of Excellence “Languages of Emotion”, Freie Universität Berlin, 14195 Berlin, Germany; 2 School of Computing and Mathematics, Plymouth University, Plymouth, PL48AA UK

**Keywords:** Action perception circuit, Cell assembly, Concept, Mirror neuron, Memory cell, Meaning, Semantic category, Semantics

## Abstract

Cognitive theory has decomposed human mental abilities into cognitive (sub) systems, and cognitive neuroscience succeeded in disclosing a host of relationships between cognitive systems and specific structures of the human brain. However, an explanation of *why* specific functions are located in specific brain loci had still been missing, along with a neurobiological model that makes concrete the neuronal circuits that carry thoughts and meaning. Brain theory, in particular the Hebb-inspired neurocybernetic proposals by Braitenberg, now offers an avenue toward explaining brain–mind relationships and to spell out cognition in terms of neuron circuits in a neuromechanistic sense. Central to this endeavor is the theoretical construct of an elementary functional neuronal unit above the level of individual neurons and below that of whole brain areas and systems: the distributed neuronal assembly (DNA) or thought circuit (TC). It is shown that DNA/TC theory of cognition offers an integrated explanatory perspective on brain mechanisms of perception, action, language, attention, memory, decision and conceptual thought. We argue that DNAs carry all of these functions and that their inner structure (e.g., core and halo subcomponents), and their functional activation dynamics (e.g., ignition and reverberation processes) answer crucial localist questions, such as why memory and decisions draw on prefrontal areas although memory formation is normally driven by information in the senses and in the motor system. We suggest that the ability of building DNAs/TCs spread out over different cortical areas is the key mechanism for a range of specifically human sensorimotor, linguistic and conceptual capacities and that the cell assembly mechanism of overlap reduction is crucial for differentiating a vocabulary of actions, symbols and concepts.

## From cognitive psychology to cognitive neuroscience and neurocomputation

**Fig. 1:**
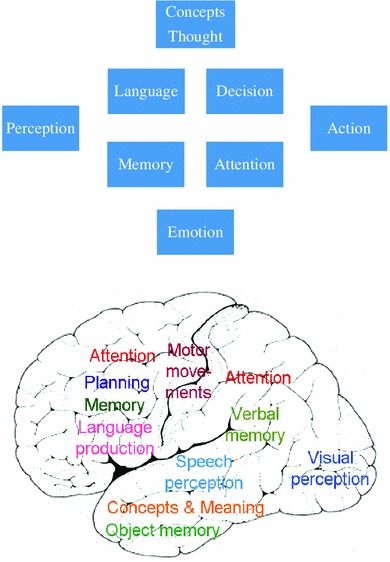
Cognitive systems and their brain areas. The *top panel* shows a range of cognitive capacities; some cognitive theories see these capacities as each being based on one or more specific cognitive (sub) systems, which work, to a degree, autonomously of the others. The *bottom panel* shows a tentative mapping of cognitive systems onto areas of cortex as it has been suggested in view of evidence from experimental neuroimaging and neuropsychological research. Note that several of the displayed mappings are under discussion (see also article text). The question of why cognitive functions are localized in one specific area (e.g. object memory in temporal cortex)—and not in a different one (e.g. occipital cortex)—is rarely being addressed

Cognitive theory specifies the subcomponents of cognition along with their interplay. These components or *cognitive systems* include modality-specific perception (visual, auditory, olfactory etc.), motor movement and action, language perception and production, attention, memory, decision, emotion, planning and conceptual thought. The separation into these systems is also manifest in the subdisciplines of cognitive and general psychology, which are devoted to these domains. These systems are seen as functionally independent to a degree, although some interaction between them is generally acknowledged. The top diagram in Fig. 1 presents a plot of major cognitive subdomains.

Cognitive neuroscience relates these mental domains to brain structures and led to proposals to map each of the cognitive modules onto one or more brain regions. Common localizations relate perception to sensory cortices, action control to motor systems, language comprehension to superior temporal and language production to inferior frontal cortex (Geschwind 1970; Price 2012). Attention is seen as a function of dorsolateral prefrontal conjoined with dorsal parietal cortex (Duncan 2006), whereas working memory may draw upon prefrontal along with inferior temporal visual (“visuospacial scratchpad”) or inferior parietal (“articulatory loop”) areas (Baddeley 2003). Decisions also emerge from prefrontal areas, where, reminiscent of a plan-generating and decision-taking homunculus, a so-called frontal executive has been postulated (for discussion, see Shallice 1982; Stoet and Snyder 2009). Concepts, the hallmark of cognition and thought, are much debated in terms of their localization in the brain; dominant views either favor a “semantic hub”, that is, an integration center for all kinds of meanings, or suggest that different semantic kinds are carried by different parts of the brain, or both (Patterson et al. 2007; Pulvermüller 2013). Functional specialization of brain areas and nuclei for different cognitive systems is evident from neuropsychological studies looking at specific cognitive impairments in patients with focal brain lesions and from neuroimaging experiments, where specific combinations of areas are found active during different cognitive tasks. The bottom panel of Fig. 1 presents some frequently discussed brain localizations of cognitive functions.

## The need for explanation

Despite all the important progress achieved in understanding aspects of cognition and in relating these aspects to specific parts of the brain, there is one important caveat immanent to most cognitive neuroscience research: The relationships between cognitive modules and brain areas are observational. An explanation is missing why cognitive process C is located at brain locus L. In Keplerian astronomy, the trajectory of Mars had been described precisely, but an explanation in terms of gravitation, mass and distance was still missing. In the same way, we can say that lesions to superior-temporal cortex lead to deficits in using language (Geschwind 1970) and that activity in dorsolateral prefrontal cortex reflects the ability to make decisions (Heekeren et al. 2008). But we do not yet understand why specific higher cognitive functions are “bound” to their specific brain loci.^1^

A meritful neurocomputational strategy filled the boxes of cognitive theory with artificial neuron-like elements in an attempt to illuminate the mechanisms underlying cognitive subprocesses carried by modules (McClelland et al. 1986; Elman et al. 1996). This important research initiative succeeded in modeling perception and aspects of language and concepts. However, it did not, in most cases, provide explanations of why particular higher cognitive functions of the human brain emerge in specific cortical fields. For example, a recent simulation study of language and conceptual processing built in a specific layer for semantic integration, which was put forward as a network correlate of ventral anterior temporal lobe, vATL;^2^ subsequently, the model simulations showed that damage to this very site leads to impairments in semantic computations performed by the model (Ueno et al. 2011). This to a degree circular strategy cannot be said to *explain* why temporal areas are so important for conceptual processing, because this knowledge is a priori implanted into the model. Rather than implanting preexisting knowledge about structure–function relationships into models, an explanatory strategy may fruitfully use information from neuroscience for deducing cortical area functions immanent to mental activity. For example, using a network implementing the general pattern of corticocortical connectivity known from neuroanatomical investigations, predictions are possible on the brain areas showing spontaneous fluctuations of activity that emerge when subjects rest and no cognitive activity is required (so-called resting states, see Deco et al. 2011). In a similar way, systematic use of known facts and principles established in cortical anatomy and physiology may guide cognitive theorizing about the specialization of local cortical functions. However, a full understanding why different brain parts are active when subjects engage in different specific cognitive tasks (see Fig. 1), why the same areas are specifically necessary for performing well on these tasks, and, more generally, why the contributions of cortical areas are so specific, has so far not been reached. To go back to the analogy: we know the Keplerian trajectories of the planets very well, but we lack the Newtonian principles for understanding the forces that keep them there.^3^

Our present approach will start from established neuroscience principles and proceed to making “predictions” about the mechanisms of cognition.^4^ Knowledge and principles revealed by neuroscience research will thus lead us to a specification of neurobiological mechanisms of cognition in terms of neuron circuits. Immanent to this strategy is our conviction that it is not sufficient for a biological theory of cognition to address the functional levels of neurons, brain areas and whole brains, but that an intermediate functional level of neuronal assemblies is necessary—similar to the level of words and sentences in between the levels of sounds and whole speeches, texts or books.^5^ This conviction is motivated by brain theory (Braitenberg 1978) and receives support from neurocomputational models implementing neurobiologically realistic auto-associative networks that mimic cortical structure. These networks tend to build cell assemblies, thus providing a brain-theoretical foundation for the formation and existence of “micro-networks” within realistic larger (brain, area) networks (Braitenberg and Schüz 1998; Braitenberg 1978; Palm 1982; Heerden et al. 1970; Wennekers et al. 2006; Garagnani et al. 2009b; Plenz and Thiagarajan 2007; Diesmann et al. 1999). The activation dynamics within the micro-networks or circuits emerging in neurocomputational network simulations provide predictions on activation dynamics in the real brain, which can either be compared with available data or be tested in future experiments. As we will explain, this approach leads to neuromechanistic explanations of a range of cognitive functions.

## Distributed neuronal assemblies: circuits for thought

We adopt the postulate that, in between the functional level of the single neuron and that of whole areas, brain systems and ultimately the whole brain, there exists a functional level of neuronal assemblies. These assemblies are strongly connected sets of neurons, which therefore each behave in a functionally coherent manner, as a functional unit. These cell assemblies are assumed to form the building blocks of cognition. This idea has a long tradition in brain theory (Hebb 1949; Braitenberg 1978; Milner 1957; Fuster 2003, 2009; Palm 1982; Abeles 1991; Plenz and Thiagarajan 2007; Singer et al. 1997; Engel and Singer 2001; Harris 2005; Ponzi and Wickens 2010; Gerstein et al. 1989). As cell assemblies may be the neurobiological vehicles of perception, action, attention, memory, decision, concepts, language and thought, they offer a perspective on cognitive theorizing that unites Marr’s (computational, symbolic-algorithmic and neuronal) levels of neurocognitive models (Marr 1982).

The cell assembly concept has been framed in different ways. Assemblies have been proposed to be situated in a small piece of brain or widely dispersed over distant areas of cortex and subcortical structures. They have been framed as functionally uniform cell conglomerates or complex circuits producing finely tuned spatio-temporal patterns when active. Their formation is commonly attributed to a form of Hebb-type learning, although, in addition to truly Hebbian synaptic strengthening by co-activation, synaptic weakening consequent to uncorrelated activation and mapping of temporal patterns by spike-time-dependent plasticity are also incorporated in recent proposals. Typically, cell assemblies are considered to be the result of learning in a structured network, whereby the structural-neuroanatomical information manifest in network structure is in part genetic or epigenetic in nature. Sensory and motor mechanisms, along with generators of internal rhythms and resultant spontaneous behaviors, are further important factors in building cell assemblies.

What makes cell assemblies especially attractive to cognitive science is the fact that they help solving a range of problems that seem to persist for other neurocomputational approaches. For example, concepts or percepts are sometimes represented as activation vectors over neuron populations (see, for example, Rogers and McClelland 2004), but it is well known that this approach runs into the problem of catastrophic overlay, making it inadequate for addressing some relevant cognitive problems (Sougné 2001; Jackendoff 2002). For example, two or more conceptual (or perceptual) activations would not result in both being active at the same time, but rather in a compromise (average or sum) vector which may be dissimilar to any of the intended representations. Sparsely coded cell assemblies with small overlap, whose activation can co-exist within the same network, offer a solution to this problem by allowing two or more representations to be active at a time. Even harder to solve is the problem of multiple instantiations, sometimes also called to “problem of two” (Sougné 2001; Jackendoff 2002): An activation vector approach can provide a putative neural correlate of a concept, but, if the network were to represent two instantiations of that concept, for example two rabbits instead of one, the vector approach fails. In contrast, a cell assembly allows for reverberatory activity to be maintained within the circuit, but, as Abeles and colleagues showed using neurocomputational simulations, such networks are not limited to storing one instantiation. If well-timed activation waves reverberate in the circuit and these waves progress with constant speed, it is possible to store information about two or more instantiations of a given engram (Hayon et al. 2005). A further exciting aspect are the perspectives this approach offers on the so-called binding problem: To store the knowledge that a green banana has been perceived, the cell assemblies for BANANA and GREEN can be coupled temporarily, for example by synchronous oscillation of the respective neuronal ensembles (Engel et al. 1992; von der Malsburg 1995; Shastri and Ajjanagadde 1993). Furthermore, cell assembly architectures have been argued to offer new perspectives for brain theory, especially in the domain of modeling language and grammar (Pulvermüller 2002; Buzsáki 2010).

As the term “cell assembly” is used in different ways by different researchers, reflecting a degree of variability of the CELL ASSEMBLY concept, it seems appropriate to spell out how we use the term in the present context (see also Braitenberg 1978; Palm 1982; Braitenberg and Schüz 1998; Pulvermüller 2002). Cell assemblies are sets of nerve cells that are “${\ldots }$ more strongly connected to each other than to other neurons” (Braitenberg 1978).^6^ The neuron members of a cell assembly do not need to be located in a small part of the brain, for example a hypercolumn, but can be spread out over different cortical areas, and even involve subcortical structures such as thalamus and striatum. Connections between different parts of the cell assembly are reciprocal in the sense that if the assembly is sliced in two parts, these parts will be connected in both directions, from part A to B and back (Braitenberg 1978). The strong linkage within the cell assembly is, in part, due to preexisting connections and, importantly, due to the correlation of activation (firing) in the past. So in this sense, the cell assembly requires preexisting neuronanatomical connectivity and is driven by specific correlated neuronal activity. We speak of “correlation” in a lose sense, with the implication that neurons that fire together strengthen their connections among each other (Hebbian “fire together—wire together” rule) and that nerve cells that fire independently of each other or even in an anti-correlated manner reduce the strength of any links they may have (‘Anti-Hebb’ “out of sync—delink” rule).^7^

These mechanisms imply that the structural properties of cell assemblies are, in part, due to functional features of their member neurons. In turn, the structural feature that cell assembly neurons are relatively strongly connected with each other has important functional implications. First, this strong internal linkage implies that the activation dynamic of a cell assembly is nonlinear; activation of a critical number of assembly neurons, the activation threshold, leads to the full activation, or *ignition*, of the whole circuit, including (not necessarily all but) most of its members. Second, after an ignition, and even if only subliminal (below threshold) activation is present in the circuit, such activity will be retained or “held” for some time, due to *reverberation* of activity, leading to hysteresis-like dynamics. Third, ignition-related explosive activation in networks requires mechanisms for activity regulation to prevent full activation of the entire network—which is reminiscent of epileptic seizures in real brains. Regulation processes are available in cortex, in the local interaction between excitatory neurons and their inhibitory local neighbors and in the area-specific interplay between cortex and subcortical structures. Such feedback regulation can be implemented as control mechanism with specific gain, which controls the “activation threshold” of excitatory neurons (and cell assemblies) in specific cortical areas (Braitenberg 1978; Elbert and Rockstroh 1987; Knoblauch and Palm 2002; Wennekers et al. 2006). Whereas reverberation may be possible in several cell assemblies at a time (with even more than one reverberation waves being present in a given assembly), the massive activation process of ignition implies that a substantial amount of inhibition is created, so that two cell assemblies that contain neurons located in the same cortical area would interfere with each other functionally, leading to mutual competition between ignition processes. The degree of competition is determined by the gain of the regulation mechanisms.

Cell assemblies in this sense imply sparse coding, that is, that, within the greater network of the cortex of the entire brain, the nerve cells that are part of, and thus significantly contribute to, one specific cell assembly represent a small minority. Estimates of cell assembly size range between hundreds and ca. 100.000 neurons, whereas the cortex contains more than $10^{10}$ (for discussion, see Palm 1993, 1990). A further feature that makes cell assemblies attractive to cognitive theory is the fact that they can overlap and be structured hierarchically. This means that two cell assemblies can be neuron sets that intersect and that one cell assembly can contain the other, thereby providing putative mechanisms for relationships between concepts and meanings (Pulvermüller 2002; Wennekers 2009).

We will also use the words “distributed neuronal assembly (DNA)” or “thought circuit (TC)” when speaking about cell assemblies in the sense explained in this section. In the next paragraphs, we will now outline a model of DNA/TCs, highlighting both cell assembly mechanisms and their role as carriers of cognition.

## Action and perception

Sensory stimulation causes activity in primary sensory fields from where activity propagates to secondary modality-preferential and higher multimodal areas. Given a reasonably high signal-to-noise ratio, a repeated sensory event therefore activates similar populations of sensory cortical neurons repeatedly and the frequently co-activated neurons may therefore strengthen their mutual connections and merge into a circuit. The perception and recognition of objects known from sensory experience may be based on the ignition of such sensory cell assemblies. Neurocomputational work implementing established neuroscience principles has shown that repeated local firing gives rise to local cortical circuits including many neurons (Doursat and Bienenstock 2006).

In contrast to local and purely uni-modal sensory activation, motor movements and actions are always characterized by both sensory and motor information. As actions are brought about by neuronal activity in the motor cortex and adjacent premotor and prefrontal areas, repeating similar movements will therefore lead to the formation of motor circuits. However, as self-produced movements imply perception of aspects of these actions, motor circuits normally do not stand alone. Given there is concordant auditory, visual and somatosensory input—for example when hearing and feeling oneself articulate the syllable “ba”, or when feeling, seeing and hearing the hammer in one’s hand strike—there is correlated activity in different motor, sensory and multimodal brain areas (Braitenberg and Pulvermüller 1992; Pulvermüller 1999). Given there are sufficiently rich long-range cortico-cortical connections between these areas, the correlated multisite activation pattern leads to the formation of widely distributed neuronal assemblies that bind motor and sensory aspects of a specific action. These action perception circuits (Pulvermüller and Fadiga 2010) are a special type of cell assembly in that they include neurons in different areas distant from each other (e.g., auditory and motor cortex) and exhibit a specific distribution over the cortex that reflects, to a degree, the sensory and motor type of information stored (e.g., articulatory and auditory, but not hand motor and visual,^8^ in the case of articulations, vice versa in the case of hand waving). As motor neurons are included in these action perception circuits, performance of specific motor commands and muscle contractions can be brought about by their ignition, and, as specific sensory cells are also included, the perception of sensory aspects leading to the recognition of the same type of action performed by others is equally based on the ignition of the same action perception circuits.

Action perception circuits conceived as distributed neuronal assemblies establish a functional link between specific sets of sensory and motor information. As such, they provide a mechanism for what Braitenberg and Schüz consider the primary function of the neocortex, namely *information mixing*, that is, the joining together of information across modalities (Braitenberg and Schüz 1998), which may be especially important for language processing (Braitenberg and Pulvermüller 1992; Braitenberg and Schüz 1992). As information about an action and its corresponding perceptions are joined together in an action-specific manner, action perception circuits also provide a mechanism and explanation for *mirror neurons*. Mirror neurons are cells in the premotor and inferior parietal cortex that respond both during performance and during perception of one type of action (for example grasping, Kohler et al. 2002; Rizzolatti and Craighero 2004). In the articulatory motor system, mirror activity has also been reported for specific speech sound types (Fadiga et al. 2002; Watkins et al. 2003; Pulvermüller et al. 2006). The correlation learning principle together with the known cortico-cortical connectivity between relevant sensorimotor areas of cortex provides an explanation why action perception circuits emerge and, thus, why mirror neurons exist. In neurocomputational studies, mirror neuron activity could indeed be shown as a consequence of Hebbian-associative learning between actions and corresponding perceptions (Garagnani et al. 2007; Hanuschkin et al. 2013). Furthermore, recent experiments in humans showed that mirror activity is indeed tied to aspects of action perception learning and therefore substantially strengthens this position (Pulvermüller et al. 2012; Giudice et al. 2009; Engel et al. 2012). It might therefore seem appropriate to consider mirror mechanisms not as fundamental, but, instead, as a consequence of the more basic neuroscience principle of correlation learning and of cortical connectivity. We note, however, that for mirror activity already observable very early in life (Lepage and Theoret 2007; Yeung and Werker 2013), further research is necessary to address the question whether correlation learning might have contributed also in these cases and to what degree genetic or epigenetic mechanisms are necessary to bring it about. Crucially, information mixing and mirror neuron activity cannot be explained by associative learning alone (cf. Heyes 2010), but requires preset specific cortico-cortical connectivity between relevant sensory and motor areas, which provide the substrate for such mirror learning. This point will be of special relevance in the language section below where a connectivity difference between macaca and human is related to vocabulary learning ability.

**Fig. 2:**
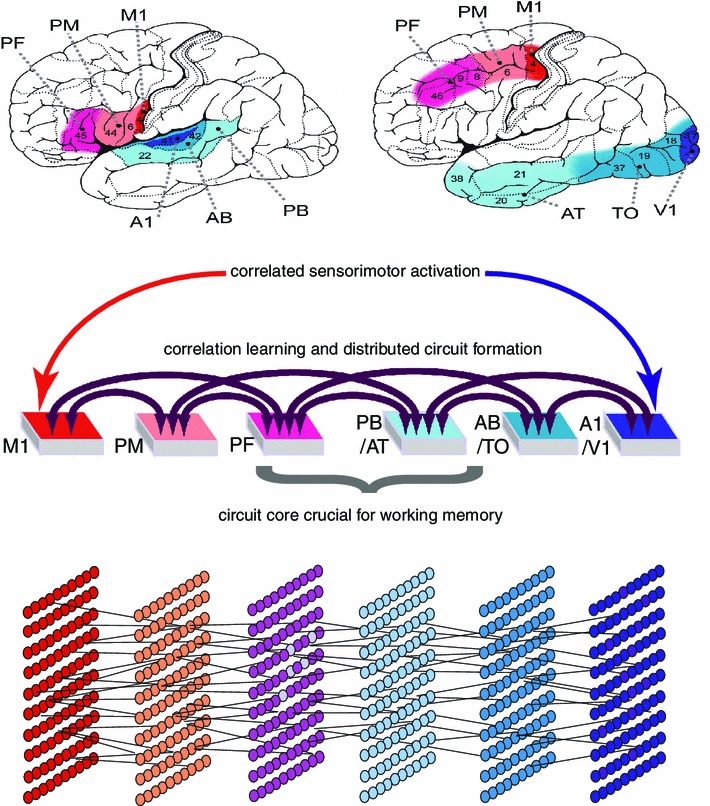
Mechanisms of the formation of action perception circuits, that is, distributed cell assemblies (DNAs) interlinking information about actions and concordant perceptions. The *top panel* on the *left* shows areas involved when mapping the articulatory motor schema of a spoken word and the sounds brought about by such an articulation: primary motor (M1), premotor (PM), prefrontal (PF), auditory parabelt (PB), auditory belt (AB) and primary auditory (A1) cortex. The *top right panel* shows areas relevant for mapping finger movements to visual stimuli: apart from more dorsal frontal areas, anterior temporal (AT), temporooccipital (TO) and primary visual cortex (V1). The *middle panel* shows the connection structure of these areas, which is similar for the auditory-articulatory and visuomotor domains. *Middle panel*: Functionally, correlated activity in primary areas drives formation of action perception circuits but core parts of these cell assemblies develop in higher (PF, PB, AT) areas because of corticocortical connection structure. Core parts are most important for memory and decisions. The *bottom panel* illustrates the distributed character of the cell assemblies, which interlink neurons in all relevant areas but still function as one closed information processing device and representational unit

For determining the cortical distribution of DNAs, both functional neuroscience principles (correlation learning) and structural knowledge about specific neuroanatomical connectivity are equally relevant. The neuroanatomical connections between primary sensory and motor cortices are not direct in most cases. With the exception of the link between primary motor and somatosensory cortex, which lie side by side and are directly linked, connections between primary areas are indirect, traveling through adjacent “secondary” modality-preferential cortices and further multimodal sites adjacent to these (Fig. 2, middle panel). This connection structure implies that action perception circuits incorporate neurons in all of these “switch over” regions through which sensorimotor activation waves must travel to make contact with each other. In a model of action perception learning that incorporates correlation learning mechanisms and information about neuroanatomical connectivity, cell assembly formation was found as a result of co-activation of neural elements in primary motor and sensory “areas” of the network (Fig. 2, bottom panel, Garagnani et al. 2007, 2008, 2009b). The learning of correlations between articulatory movements and auditory input patterns—as it is present when infants “babble” and use their first words (Locke 1993)—led to cell assemblies distributed over the model correlates of inferior prefrontal, premotor and primary motor cortex in inferior frontal cortex as well as primary auditory, auditory belt and parabelt areas in superior temporal cortex. Likewise, learning the co-occurrence of hand movements and specific object shapes and colors was manifest in cell assembly formation across primary and inferior temporal visual processing areas along with dorsolateral prefrontal, premotor and motor cortex (Garagnani et al. 2007, 2009b; Garagnani and Pulvermüller 2013). Contrary to the intuition that it might be difficult to separate and specifically activate cell assemblies (Milner 1996), correlated sensorimotor patterns repeatedly presented to networks with neuroanatomically inspired area structure and sparse random connectivity led to cell assemblies with small overlap that could be defined using robust criteria related to activation dynamics (Garagnani et al. 2009b).^9^

At the cognitive psychological level, the ignition of a cell assembly provides a mechanism for the *recognition *of a familiar stimulus that matches a cortical representation. It also provides a mechanism for the recognition of familiar stimuli based on insufficient information—for example an object partly hidden behind a different one or an incomplete spoken word. Note that spoken words are normally being recognized before they end; even if alternative word candidates match a given input fragment, the more frequent item may indeed be preferred in the absence of unambiguous evidence (Marslen-Wilson 1990). The role of frequency as a facilitatory factor in the recognition process is explained by the Hebbian learning mechanisms and the resultant stronger links and more robust activation dynamics of the more frequently activated DNA/TCs.

## Memory

Cell assemblies are structured internally. Contrary to the suggestion that such circuits are just an amorphous agglutination of neural elements, it has been argued that they can be separated into a most strongly connected core or *kernel* part and a periphery or *halo* (Braitenberg 1978). In the ignition process, the entire cell assembly becomes active, including core and halo parts, whereas the most strongly connected core will naturally form the basis for reverberant activity. Whereas ignition processes provide the mechanism for the recognition of a familiar stimulus, the sustained reverberation of activity represents a mechanism for maintaining information active in memory, so-called active or working memory (Fuster 1995; Zipser et al. 1993).

Crucially, there are riddles surrounding the brain organization of perception, motor and memory processes that have not been solved in spite of efforts in current neuroscience and cognitive research. Perception, action and memory tend to dissociate. It has been suggested that “object knowledge is stored as a distributed network of cortical regions and that the organization of these regions may closely parallel the organization of sensory, and perhaps also motor, systems in the human brain” (Martin et al. 1995). In contrast to such an “embodied” sensorimotor view on knowledge and memory, it is well known that memory storage of specific sensorimotor knowledge most typically and most strongly engages the dorsolateral prefrontal, anterior temporal and other higher multimodal cortices. These areas are not part of dedicated sensory or motor systems, but rather represent multimodal cortices. The suggestions that “embodied” action- and perception-related information in sensorimotor cortices drives the formation of cortical cell assemblies therefore seem in contrast to the observations from neurophysiological research on working memory: The most substantial and sustained reverberating memory activity is known to be present in multimodal areas such as dorsolateral prefrontal cortex and anterior temporal lobe, where cortical cooling also impairs memory specifically (Fuster 1995). Therefore, motor and sensory processes and memory dissociate from each other at the neurofunctional level and a neurocognitive theory need to explain why this is so.

Such an explanation is possible based on the distinction between kernel and halo of cell assemblies, along with information about corticocortical connectivity (Pulvermüller and Garagnani 2014). Most neurons included in the core part of the cell assembly that is most relevant for maintaining reverberation, and therefore for working memory, are located in those brain areas between which the most elaborate connectivity is present. Comparing the connectivity structures interlinking the modality-preferential primary and secondary areas and the multimodal convergence areas, it appears that the latter show the largest number of cross-area links (for discussion of neuroanatomical evidence, see Garagnani and Pulvermüller 2013). For this reason, they provide the substrate for (most of) the most strongly interlinked assembly cells and hence for building cell assembly cores.^10^ Therefore, the core part carrying reverberant active memory processes is in association with cortices and thus outside both sensory and motor regions. Due to specific auditory-motor and visual-motor connectivity pathways, verbal memory processes primarily draw on superior temporal/temporoparietal auditory and inferior dorsolateral prefrontal cortex. Contrasting with the relevance of multimodal areas for working memory, motor regions are most crucial for acting—as their damage not only partly lesions the cell assembly but, in addition, disconnects it from its motor output—and sensory regions are most crucial for perception—as their damage disconnects it from sensory information input. Model simulations confirm that prefrontal and anterior temporal cortex are among the prime sites for working memory (Pulvermüller and Garagnani 2014; Garagnani and Pulvermüller 2013). The middle part of Fig. 2 illustrates the relationship between cortical areas, connection structure, learning driven by sensorimotor correlation and resultant memory mechanisms, using spoken words as an example.^11^ Crucially, the inner structure of DNAs can offer an explanation why the “higher” multimodal areas are so important for memory, whereas the formation of memory circuits is driven by information in the senses and the motor system.

## Decisions

From a cell assembly perspective, decisions and memory appear to be based on similar mechanisms. Reverberant memory activity lasts longest in the core part of the circuit, which exhibits the strongest average connection strength. Such high connection strength may also provide a mechanism for new activity spontaneously emerging in a circuit. Such spontaneous activation should therefore start in the circuit’s core. One reason for this local specificity lies in the fact that uncorrelated background noise activity is always present in real networks of neurons and such activity will accumulate and reverberate more efficiently in cells with multiple inputs from other noise-emitting neurons compared with less strongly interlinked ones. When first implementing networks fashioned according to cortical anatomy that gave rise to action perception circuits, we immediately observed that networks with low levels of random activity (white noise) present in all of their parts rarely stayed inactive, but that, instead, spontaneous ignitions emerged within them, without any sensory stimulation being present (see also, for example, Willwacher 1976). Such spontaneous emergence of activity in specific DNA/TCs can be seen as a putative brain mechanism of an emerging thought or *intention* to act—when activity in the cell assembly first appears, followed by a *decision *to prefer a specific action over alternatives—when the cell assembly’s activity level is substantially higher than that of its competitors, finally culminating in *performance of the action* itself—when the cell assembly fully ignites and activates its motor output. The level of activity in the cell assembly would thus represent the “stage” of an intention/decision, and the specific action perception circuit activated and its cortical distribution contain the information about the “content” of the action or perceptual decision. A range of previous research addressed the neurocomputational basis of decisions (Deco et al. 2013).

One crucial question in the emergence of decisions to act is about where such decisions come from. Why would the neuronal correlates of intentions and decisions to act be first manifest in specific cortical areas? In networks containing action perception circuits of the structure outlined in Fig. 2, spontaneous ignitions start in multimodal prefrontal and temporal areas, where activation slopes also rise most sharply toward full ignition (Garagnani and Pulvermüller 2013). The explanation of this local specificity of decision emergence can be based on background activity in the network—due to noise and previous specific activations—which accumulates most efficiently in DNA cores. Core neurons receive multiple inputs from other cell assembly neurons, more than nerve cells in the halo, which are more weakly interlinked. These multiple inputs make them most likely candidates for accumulating substantial activation if all neurons are equally subject to noncorrelated spontaneous activity (“noise”, Garagnani and Pulvermüller 2013). Activity accumulation in most strongly connected circuit parts also applies if specific informative input reaches a given circuit by way of corticocortical input, for example via connections from specific other DNAs. Therefore, activity accumulation in cores explains why and how spontaneous ignition of DNAs comes about and thus provides a candidate mechanism for the emergence of intentions and decisions to act. In this model, the factors influencing the model’s “free” decisions (the choice of one circuit over others in the spontaneous ignition process) include the accumulation of “noise” in circuits, runaway activation from previous circuit activations, specific between circuit connections and the inner connection strength and coherence of the igniting circuit itself.

Looking back at previous paragraphs, it appears that the cell assembly model implies that the neurons in the core part of DNAs, most of which are localized in higher multimodal cortices, are the primary substrate of a range of higher sophisticated cognitive mechanisms. The present section made this point for intentions and decisions and the previous one for working memory. The same cortical substrate forms the basis of working memory and for intentions/decisions, although the cortical distribution of the circuit over specific areas may differ between stimulus types, for example between words and objects. The observation that similar parts of prefrontal cortex are active in standard working memory and perceptual decision tasks sits well with these postulates (Duncan and Owen 2000; Fuster 2008, 2009; Heekeren et al. 2008). It thus appears that the question why a wide range of higher cognitive processes are carried by the same “multi demand system,” and why this network draws, especially upon prefrontal cortex (Duncan 2010), can, in part, be answered and explained by cell assembly theory.

## Attention

**Fig. 3:**
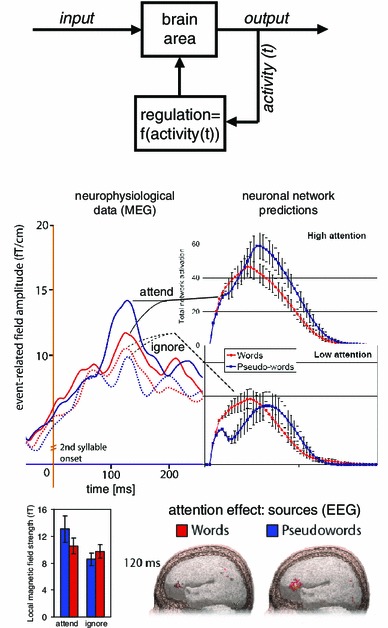
Mechanisms of attention. *Top panel*: Illustration of Braitenberg’s proposal of a *threshold regulation mechanism*, which controls activation levels in the brain or, as suggested here, in each cortical area specifically, and provides a neuronal basis for attention and task adjustment. A cybernetic feedback regulation loop controls activation in a given brain area by comparing it with a target value and feeding back a control value that depends on the discrepancy between target and actual activity (Braitenberg 1978). *Middle panel*: Attention effects on language processing as predicted by a model including threshold control and as measured experimentally. Brain responses (recorded with MEG, on the *left*) and brain model responses (*insets* on the *right*) to familiar words (in *red*) and unfamiliar pseudowords (in *blue*) when attention is directed toward these stimuli (*solid lines/inset* on the *upper right*) or away from them (*broken lines/inset* on the *lower right*). Note the great attention-related variability of responses to pseudowords and the much reduced attention effect to words (adopted from Garagnani et al. 2008, 2009a). *Bottom panels*: The significant interaction of lexicality (words vs. pseudowords) and attention is illustrated in the *bar plot* on the *left* (Garagnani et al. 2009a). The strongest cortical sources underlying the attention effects for both words and pseudowords are present in *left* inferior frontal cortex (Shtyrov et al. 2010)

As brain-like architectures are in danger of catastrophic over-activation, activation regulation processes are required (see section on DNAs above, Braitenberg 1978; Milner 1996; Braitenberg and Schüz 1998). In a similar fashion, lack of regulation and control processes makes it possible that activity extinguishes within a network. Braitenberg’s proposal of regulating the firing threshold of neurons effective in cortex generally or in an area-specific manner (see Fig. 3, top panel, Braitenberg 1978) addresses both the over- and under-activation problems and was successfully applied to neurocomputation models, where it contributes to the functionality of networks including cell assemblies (see, for example, Elbert and Rockstroh 1987; Knoblauch and Palm 2002; Wennekers et al. 2006; Bienenstock 1994). Threshold regulation can be implemented as a mechanism that provides a degree of background activity to excitatory neurons in a given area; the regulatory input is calculated as a function of previous activity of the same network part. The neuronal substrate for this feedback regulation mechanism may be provided by one of the big loops of neuroanatomical structures in which the neocortex is embedded, for example the striato-thalamic or the hippocampal loop (Braitenberg 1984; Fuster 1995; Miller 1991; Miller and Wickens 1991). A regulatory loop allows for modifying the gain or amplification factor of the regulation function, which influences the speed and power of regulation. A range of recent neurocomputational simulations successfully used background activity (noise) to prevent extinguishing of network activity together with area-specific inhibitory feedback regulation to prevent several ignitions at a time and to control the degree of competition between partly active reverberating cell assemblies (Bibbig et al. 1995; Palm and Sommer 1995; Knoblauch and Palm 2001; Wennekers et al. 2006; Garagnani et al. 2008).

At the cognitive level, the degree of inhibition in a neural and cognitive system has been related to attention. According to the well-established *biased competition model* (Duncan 2006), attention to objects or loci in space is controlled by two main factors, the degree to which there is a bias toward them and the degree to which different putative targets of cognitive processing compete with each other. In cell assembly networks, the degree of preactivation or priming of a circuit (that is, its level of preexisting activity) provides the neuromechanistic correlate for the bias and the degree of regulatory inhibition (or threshold regulation) underpins competition (for discussion, see Garagnani et al. 2008).

Adopting these neurocognitive mechanisms for attention, different levels of attention can be simulated in brain-like neurocomputational networks by choosing different levels of regulatory inhibition. These different regulation levels provide different levels of inhibition between DNAs; in addition, threshold regulation can be chosen so that it guarantees that, within given areas, only one circuit ignites at a time and that each ignition is followed by strong global inhibition (see Section on DNAs). Now, the level of attention can be adjusted by altering regulation gains. High attention and availability of ample attentional resources can be implemented by low degrees of regulation and inhibition. With low inhibition/high attention, several cell assemblies can simultaneously become partly active. (Note that, for guaranteeing functionality of the cell assembly network, it may still be advantageous to keep inhibition constants within a range preventing more than one ignition at a time within a given area.) This corresponds to the situation when an incomplete word (such as “wai...”) is perceived and several lexical alternatives from its “cohort” are partly activated (“wait”, “wail”, “waif” etc., see Marslen-Wilson 1987). Therefore, with high attention, ample availability of “cognitive processing resources” and thus weak inhibition in the network, the alternative lexical circuits can co-activate and each create some substantial activity. In contrast, under low-attention conditions, when cognitive processing resources are very limited, and therefore, strong inhibition is present in the network, the competing cell assemblies would only be allowed a much reduced activation level (i.e., the input “wai” may only minimally activate some members of its cohort). In contrast to these strongly attention-dependent processes for meaningless unfamiliar stimuli (pseudowords) or ambiguous word fragments, the presentation of a meaningful word leads to the ignition of its corresponding stored cell assembly which, regardless of attention level, leads to the suppression of competitor circuits. In this case, a modulation of the gain of feedback regulation would have a minor effect on cortical activation dynamics because it is dominated by the ignition process, which takes place in spite of threshold regulation.

These predictions were supported by neurocomputational simulations using a model of the language cortex with noise background activity and attention modulation implemented in the gain of area-specific inhibitory regulation processes. Word patterns, which had previously been learned by the network, resulting in word-specific action perception circuits, indeed led to network activation, which was only modulated to a small degree by the level of feedback inhibition and attention. However, pseudoword stimuli, which had not been learned previously thus lacking corresponding cell assemblies, led to simultaneous partial activation of word circuits, which massively varied with the level of the gain and thus attention (Garagnani et al. 2008). Critically, the same differential pattern of attention effects on word and pseudoword processing was found in novel experiments performed with human subjects and recordings of EEG and MEG responses. These results provide strong support for the model’s predictions (Fig. 3, middle panel, Shtyrov et al. 2010; Garagnani et al. 2009a).

The attention model of cell assembly competition makes one further critical prediction: attention should not have one single general brain manifestation and locus but should instead always occur within the range of cortical areas where competing cell assemblies are located. In the case of visuospatial decisions, this may be the dorsolateral prefrontal cortex along with lateral parietal areas. However, in the case of spoken language, these are the multimodal areas in the perisylvian cortices including inferior frontal, inferior parietal and superior temporal subregions. Model simulations further indicate that in case of sensory simulation of cell assemblies that link action and perception information, attention effects are most pronounced in their frontal, action-related network parts. This explains why attention effects in spoken language processing are most clearly manifest in Broca’s region in left inferior frontal cortex and not in dorsolateral prefrontal cortex (Fig. 3, Shtyrov et al. 2010), and why lexical competition effects are manifest in this area as well (Thompson-Schill et al. 2005).

## Language

Language can be seen as a huge set of meaningful elements (usually tens to hundreds of thousands) that can be combined to yield even larger sets of constructions. Each construction can be used to perform specific communicative actions, which follow each other according to further combinatorial schemas. The purpose and intention related to a communicative action is intrinsically related to the typical sequence schemas it is part of. For example, a request is characterized by the desire or intention to obtain something, which is also manifest in the typical sequence that a request is frequently followed—not always of course—by the action of transferring the requested entity to the requesting party. Therefore, combinatorial mechanisms for language need to be implemented at different levels, at the level of sounds following each other in words, at that of morphemes and words connected in sentences and texts and at the level of social-communicative actions interlinked with each other by pragmatic rules and hierarchical action schemas.

In the cell assembly framework, the phonological sequences according to which speech sounds are lined up in words can be implemented by synfire chains—parallel and precisely timed neuron chains contained in cell assemblies (Abeles 1991; Braitenberg and Pulvermüller 1992). Note that explicit modeling of such spatiotemporal patterns these chains generate is challenging and explicit neurocomputational work using realistic circuit architectures incorporating major aspects of the anatomy of the language cortex are still missing; such models, which may build on established computational work on synfire chains (Diesmann et al. 1999; Hayon et al. 2005; Verduzco-Flores et al. 2012), represent an important target for future research. Syntactic combination can be implemented neuromechanistically by direct and indirect links between cell assemblies along with activation dynamics present within memory-active cell assemblies (Pulvermüller and Garagnani 2014; Pulvermüller 2010; Buzsáki 2010). For example, indirect between-assembly links by way of additional combinatorial neuronal assemblies may interconnect groups of lexical circuits—corresponding, for example, to the combinatorial relationship between syntactic–semantic categories of nouns and verbs (Pulvermüller and Knoblauch 2009; Humble et al. 2012). Likewise, combinatorial relationships between communicative actions can be implemented by sequential and hierarchical links between cell assemblies backing social-communicative actions (Egorova et al. 2013; Pulvermüller et al. 2014). Recursion, the repeated application of a combinatorial schema at the level of word or communicative action sequences, may require multiple cell assembly activation along with storage of the temporal relationship of their activation times, which, arguably, may draw upon the effect that, after an ignition, activation levels fall off monotonously for some time so that the sequence of activations can be stored in activation hierarchies (Pulvermüller 1993; Buzsáki 2010). Using the neurobiological mechanisms for recursion and embedding summarized here, abstract linguistic grammars can be rewritten in terms of algorithms denoting cell assembly architectures and their dynamics (Pulvermüller 2002; Wennekers and Palm 2009).

Although in linguistics the combinatorial properties of language are usually emphasized as the most important aspect of human language, the sheer quantity of words and stored meaningful forms immanent to human languages already sets them apart from all known animal communication systems. A neurobiological correlate for such huge vocabularies seems obvious (see also section on action and perception): The left perisylvian cortex, where correlated motor and sensory activity are present during articulation of words, is more strongly connected by way of dorsal long-distance connections through the arcuate fascicle in humans compared with nonhuman monkeys, and it tends to be more strongly developed in the left hemisphere than in the right (Rilling et al. 2008; Catani et al. 2005; Dubois et al. 2009; Buchel et al. 2004). Building a huge vocabulary may critically depend on the availability of rich frontotemporal connectivity (although such connectivity is likely not sufficient, see below). If so, the following critical experimental predictions result: Listening to unfamiliar novel words may only activate auditory cortex, and perceptual learning of such word forms may not change this. However, active repetition of novel word forms by the learner leads to frontotemporal correlation of neuronal activity, which is mapped most efficiently in the left hemisphere of most individuals because of the predominance of frontotemporal connections there. Therefore, left lateralized perisylvian cell assemblies develop as a consequence of articulatory learning of novel word learning by repetition. These cell assemblies should include left inferior frontal neuron sets.

Experimental study of word learning indeed showed left lateralized inferior frontal activation during passive listening to novel word forms after articulatory but not after perceptual learning. These results support a link between vocabulary build-up and action perception circuit formation by way of human-specific left lateralized frontotemporal connections. The cell assembly perspective suggests that establishing the rich cortico-cortical connections in left perisylvian cortex was humans’ key step to language. Although rich connections in themselves cannot explain huge vocabularies, they may still represent a necessary feature of such an explanation.

Direct connections between word-related circuits cannot explain why combinatorial principles apply to large word classes and sometimes lead to the production of constructions the speaker has not encountered before. This phenomenon of generalization—together with that of recursion—led linguists to argue that much grammatical knowledge must be immanent to the genetic code. Further explanation would therefore not be necessary. However, there is information in the recombination structure of words in sentences. For example, subject nouns tend to co-occur with predicate verbs, determiners with nouns and adjectives with nouns. A word group A may form, because its members $\hbox {a}_{1},\hbox { a}_{2}, {\ldots }\,, \hbox {a}_\mathrm{m}$ tend to co-occur with members $\hbox {b}_{1},\hbox { b}_{2}, {\ldots }\,, \hbox {b}_\mathrm{m}$ of word group B. It would not be economic to assume that the species stores information in the genetic code the individual can get for free by mapping correlations in the input.

**Fig. 4:**
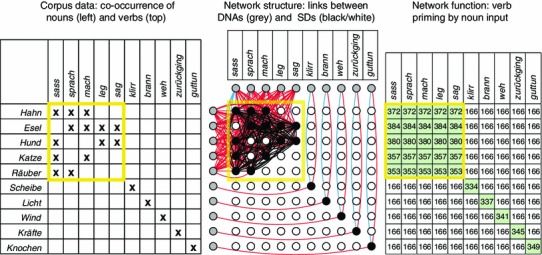
Combinatorial learning of noun-verb co-occurrences in an auto-associative neuronal network model. *Left panel:* The matrix shows word pair co-occurrences in a mini-corpus that served as input to the network (verbs in top row, nouns in left column; crosses indicate co-occurrences in text). The matrix section of frequent recombination is highlighted in yellow. *Middle panel:* Neuronal elements, DNAs, for the same words (grey circles), sequence detectors, SDs, sensitive to specific word pair sequences (white and black circles in square arrangement), and connections between them. Black SDs indicate learning of specific sequences of nouns and verbs previously presented to the network. Note that the DNAs of all words previously involved in combinatorial exchanges are interlinked by way of a conglomerate of heavily interconnected sequence detectors, the combinatorial neuronal assembly (black SDs and black between-SD links on top left). Formation of general links between those nouns and verbs, which frequently occur in combination with the respective other word group (yellow square), by formation of the combinatorial neuronal assembly is a neuromechanistic result of co-activation of some (not all) of the relevant SDs. *Right panel:* Result of combinatorial learning for network functionality. After learning, activation of any noun involved in the combinatorial schema (yellow square) primes all of the verbs involved to the same degree, regardless of whether the specific word sequence itself had been subject to learning. The dynamics are discrete in the sense of an all-or-none response. Note the generalization to not previously encountered sequences. Lexical items not participating in the combinatorial exchanges are not bound into the combinatorial neuronal assembly (modified and adopted from Pulvermüller and Knoblauch (2009))

The nervous systems of various animals, from insect to mammal, house vast numbers of neurons that are sensitive to the direction of a visually perceived movement, and the mechanism behind this direction sensitivity includes the detection of the order of the activations of neurons, that is, sequence detection (Hubel 1995; Reichardt and Varju 1959; Barlow and Levick 1965). It does not appear as a particularly strong assumption that the human brain can do with words what a vast range of nervous systems can do with visual stimuli: to detect in which order they appear. In this sense, there may be neuronal elements that respond specifically to sequences made up of two words. For the sequence of word-$\hbox {a}_\mathrm{i}$-followed-by-word-$\hbox {b}_\mathrm{j}$, there would be a sequence detector $\hbox {SD}_\mathrm{ij}$ responding specifically to this sequence of ignitions. If sequence $\hbox {a}_\mathrm{i}$-followed-by-$\hbox {b}_\mathrm{j}$ appears, there is strengthening of connections between the circuits for words $\hbox {a}_\mathrm{i}$ and $\hbox {b}_\mathrm{j}$ by way of the $\hbox {SD}_\mathrm{ij}$ circuit.^12^ Suppose the words $\hbox {a}_\mathrm{i}$ and $\hbox {b}_\mathrm{j}$ have each already appeared in several different contexts before and therefore their cell assemblies acquired strong connections to a range of other SDs; in this case, a new word sequence will co-activate its specific SD together with the other SDs strongly linked with the cell assemblies of $\hbox {a}_\mathrm{i}$ and $\hbox {b}_\mathrm{j}$. Thus, a number of SDs will be co-activated and, assuming there is auto-associative connectivity within the SD network, bound together by way of neuronal learning mechanisms. We have previously shown that such interlinking of co-activated SD circuits provides a mechanism for combinatorial generalization (Pulvermüller and Knoblauch 2009; Knoblauch and Pulvermüller 2005). The combinatorial neuronal assemblies, which function as discrete elements that ignite and reverberate, link together word groups that need to be defined at both the lexical and semantic levels, such as animal nouns and action-related verbs. Crucially, the discrete combinatorial neuronal assemblies provide a mechanistic explanation for combinatorial generalization (as illustrated in Fig. 4). Such generalized combinatorial learning can be effective at the level of individual word forms, and at that of larger constructions and social-communicative action schemas (speech acts) as well.

We submit that cell assemblies may help explain why and how combinatorial learning comes about. Links within and between cell assemblies may be essential for combinatorial mechanisms at the linguistic levels of speech sound sequences, word combinations and hierarchies in constructions, and social-communicative action schemas.

## Concepts and meaning

**Fig. 5:**
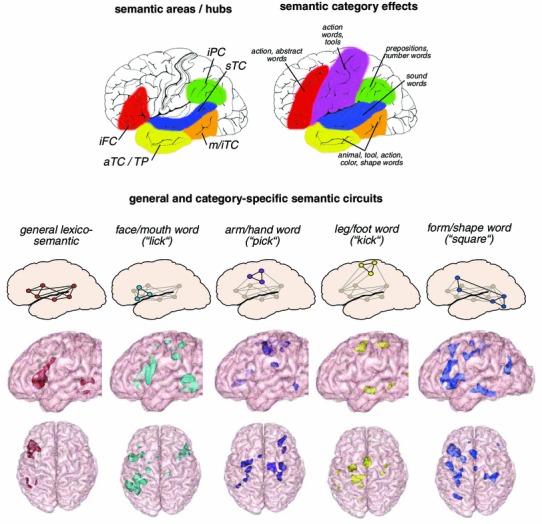
Semantic brain mechanisms. *Top left panel*: Areas of particular importance for general semantic processing as proposed in the literature. *iFC* inferior frontal cortex, *iPC* inferior parietal cortex, *sTC* superior temporal cortex, *m/iTC* middle/inferior temporal cortex, *aTC* anterior temporal cortex, *TP* temporal pole. *Top right panel*: Cortical areas where *semantic category specificity* was reported in neuropsychological patient studies and neuroimaging research—for word categories semantically related to actions (for example, “grasp”), numbers (“seven”), space (prepositions, e.g., “under”), sound (“bell”), color (“*green*”), shape (“*square*”), animals (“cat”), tools (“knife”) and abstract entities (“love”, “beauty”). *Middle panel*: Model of general lexico-semantic circuits shared by all word types (leftmost graph) and category-specific circuits for four different semantic word types (from *left to right*: face-related, arm-related and leg-related action words, form-related word). *Bottom panel*: Brain activation for the same types of words as revealed by fMRI experiments and cluster analysis (adopted from Pulvermüller 2013)

Where does the brain store and process meaning? Figure 1 (bottom panel) graphically summarizes one popular answer to this question that concepts are placed in the “semantic hub” in ventral anterior temporal lobe (Patterson et al. 2007). However, this and similar statements actually obscure the fact that there is, in fact, a wide variety of opinions. Different researchers postulate semantic-conceptual centers, semantic binding sites or “hubs” in inferior prefrontal (anterior area of Broca and next to it) (Bookheimer 2002), inferior parietal (angular gyrus) (Binder and Desai 2011), superior temporal (Hillis et al. 2001), temporopolar (Patterson et al. 2007) and/or inferior to middle posterior-temporal (Hickok and Poeppel 2007) cortex (see Fig. 5, left panel, and, for review, Pulvermüller 2013). The wide distribution of these areas is consistent with the idea that, in order to represent concepts, information from many senses must be made available and be integrated with each other (Lichtheim 1885; Wernicke 1874).

In addition to the wide cortical distribution of “semantic areas” (Binder and Desai 2011; Pulvermüller 2013), a most exciting aspect of semantic processing in the human brain is that many brain lesions affect semantic categories to different degrees, suggesting that brain areas specialize in specific semantic content (Fig. 5, right panel, Shallice 1988; Warrington and McCarthy 1987). Why should this be so? Why is there, apparently, a *semantic topography *whereby animal and tool words (such as “camel” and “hammer”) draw on different temporal and frontal areas, action concepts (“grasp”) engage the motor system, prepositions (“under”) involve the inferior parietal lobe, sound words (“bell”) rely on auditory cortices and even olfactory (“cinnamon”) and gustatory words (“salt”) spark activation in piriform and anterior insular cortex (Allport 1985; Pulvermüller 1999; Binder and Desai 2011; Pulvermüller 2013)?

An explanation of semantic topography and category specificity is possible in terms of correlation learning mechanisms in association with corticocortical connectivity. Lesions in, and stimulation to, the motor system have a causal effect specifically on the processing of action-related words, because their word forms are linked by correlated application to action schemas whose cortical mechanisms include neuronal populations in the motor areas of the human brain. The explanation assumes that, at least for some typical action words learned early on in childhood, word form processing occurs in close temporal contingency with activation of a motor program realized as a circuit reaching into motor areas. Therefore, a higher-order circuit develops by way of associative correlation learning that unites the representation of the word form and that of the motor program into one higher-order DNA. At the cognitive level, higher-order DNAs provide the cortical “representation” and the mechanism for the processing meaningful words, that is, the word form together with its semantics, which, in the case of an action word, includes knowledge of the motor schemas the word is typically used to speak about.^13^ This consideration motivates the use of the term “thought circuit” (TC) for this type of DNA. Stimulation of the motor part of this thought circuit can subsequently influence the processing of the action-related word, and removal of the motor part will reduce the overall feedback in the circuit thus yielding a processing disadvantage (Neininger and Pulvermüller 2003; Pulvermüller et al. 2010, 2005; Liuzzi et al. 2010; Willems et al. 2011; Shebani and Pulvermüller 2013).

Similar points can be made for other semantic word types (Pulvermüller 1999). Please remember, for example, the above mentioned findings that, odor words such as “cinnamon” activate in primary olfactory cortex and anterior insula (González et al. 2006) and sound words such as “bell” draw especially on auditory areas in superior temporal cortex, where lesions affect the sound word category most severely (Kiefer et al. 2008). The higher-order DNAs or TCs bound together by long-distance corticocortical fibers include word form circuits in perisylvian cortex, category-specific parts of the semantic circuits reaching into modality-preferential areas and connection hubs in frontal, parietal and inferior temporal cortex interlinking the latter. It appears that the principle of correlation learning helps to explain a good deal of category specificity of concepts and aspects of the “semantic topographies” of words that relate to specific types of concepts (bottom panel of Fig. 5), along with the involvement of general semantic hub-like convergence areas. It remains to be seen whether alternative approaches can provide alternative explanations of category-specific semantic topographies in the cortex.^14^

## Why are humans special?: A cell assembly hypothesis

The cell assembly perspective leads to a range of novel explanations of localizations of higher human brain functions. We believe that such explanation represents a crucial step in advancing cognitive neuroscience from a descriptive (Keplerian) to an explanatory (Newtonian) science. In previous paragraphs, we discussed explanations of the localization of specific cognitive functions, including memory, decision, attention, language and concepts. Over and above this so-called *where-question* (see, for example, Pulvermüller 1999), there are other types of explanations, many of which are still missing in cognitive neuroscience. In the section on language, we already touched upon the questions of combinatorial generalization and vocabulary size. Nevertheless, even after these elaborations, we still lack a full answer to many questions about the sheer size of human cognitive repertoires: Why do humans, but not other species, develop an exquisite vocabulary of so many words? Likewise, why do humans have an over-rich action repertoire at their hands, which their nearest neighbors lack? How come that, apparently, humans are able to make fine-grained conceptual distinctions that are modified by learning and can even influence the individuals’ perception according to categorical boundaries immanent to the concepts? These questions call for answers in neuromechanistic terms. In the remaining paragraphs, we will elaborate on one type of answer offered by cell assembly theory.

Accounts in terms of multiple modular cognitive systems offer the possibility to explain the abovementioned fine cognitive capacities, which make humans special, as epiphenomena of an exquisitely capable neural substrate that brings to fruit the processing potential of each of its separate composite systems or cortical areas. The key may therefore be an as yet not discovered feature of local neuroanatomical structure and/or neurophysiological processing that makes the areas of the human cortex special and allows each of the individual cognitive systems to work more efficiently. This approach may still have difficulty explaining why these human features might have arisen in one go, with apes and monkeys lacking all the mentioned superb abilities, whereas all (unimpaired, not deprived) humans apparently share them. Wouldn’t it be more plausible that a single structural or functional feature developed first in one area and then spread gradually across others? In this case, one might expect intermediate forms, for example a monkey with monkey-like conceptual distinctions but huge vocabulary, but such intermediate forms do not seem to exist.

A second approach might therefore be the emergence of one specific brain area (or small set of brain areas) that is special and provides humans with their specific higher abilities. The *multiple demand network*, especially its prefrontal part, is the prime candidate in such an account, where, by hypothesis, the parts of complex mental programs are being separated, organized and controlled (see also the section on decisions, Duncan 2010). However, a multiple demand network in prefrontal cortex does not explain the large vocabularies in the action, perception and language domains. These large vocabularies seem to depend on specific brain areas, including perisylvian cortex and motor systems, which show little overlap with the multiple demand system.

The cell assembly account suggests that the key to human cognition lies in the *connections between cognitive systems, in their structural linkage and functional interaction*. Such interlinkage would solve a key problem at the neurocomputational level, which arises whenever fine-grained distinctions need to be implemented in neuronal networks. From a neuromechanistic perspective, it is difficult to functionally separate circuits that share much of their neuronal resources. If two cell assemblies overlap to a large degree, they tend to merge functionally (Palm 1990, 1993). If one is activated, activity spreads over to the other and both together ignite. The best way to avoid this problem is to build architectures in which circuits only overlap to a small degree. One cell assembly may intersect with many others, but, in order to separate circuits functionally, each intersection should be small relative to the size of each cell assembly (Palm 1990, 1993). The key to human cognition may therefore lie in *overlap reduction in cortical circuits*.

**Fig. 6:**
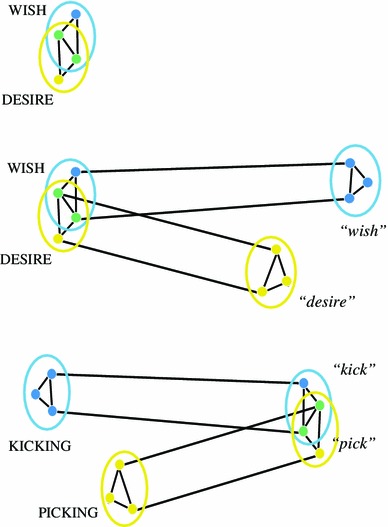
Overlap reduction in distributed neuronal assemblies or thought circuits (DNAs/TCs) as a mechanism for fine-grained cognitive discrimination. The *top panel* illustrates the overlap and functional separation problem for the concepts WISH and DESIRE, which are semantically very similar so that their cell assemblies overlap substantially and therefore activate each other and are difficult to separate functionally. It is unclear how a conceptual network can separate such conceptual circuits. *Middle panel*: Overlap reduction can be achieved by binding each circuit with an entirely different circuit, by cross-modality transcortical linking. Similar concept circuits are linked to dissimilar word form circuits; therefore, the neuronal overlap of the entire TCs is being reduced. *Bottom panel*: The mechanisms work both ways, also the circuits of similar word forms can be made more distinct by way of conceptual links. Overlap reduction in DNAs/TCs provides a mechanism for fine-grained conceptual, linguistic, perceptual and motor discrimination and may be key to human cognition

How is it possible to prevent large overlaps? In case of the representation of very similar concepts—for example the concepts of WISH and DESIRE—the overlap of semantic features is huge.^15^ Most attributes that characterize one of the entities are also characteristic of the other, although there are few fine-grained differences, for example, a DESIRE can be seen as especially strong and long lasting. In a neuronal theory assuming correspondence between conceptual features and neuronal elements, the brain representations of such similar concepts would therefore overlap substantially. The cell assemblies of similar concepts would thus share most of their neurons, with only few neuronal elements specific to each conceptual circuit. This situation is illustrated schematically in the top panel of Fig. 6.

In order to functionally separate the concepts, the neuronal circuits would need to become more distinct. In other words, their intersection would need to become smaller relative to each cell assembly’s overall size. Shrinking the overlap area does not seem to be an option, especially as neuronal elements in the overlap area would be most strongly connected with each other due to their frequent co-activations when either of the concepts is being processed. It appears as the best strategy to add distinct neuron sets to each of the conceptual circuits.

Relative shrinking of the overlap area can be achieved by enlarging the distinct parts of cell assemblies. One way of implementing this is by adding specific neuronal elements to each assembly by correlation learning. In the case of the concepts WISH and DESIRE, which are very similar in terms of semantic features, each of the conceptual circuits could be linked arbitrarily to maximally dissimilar symbols or word forms, whose neuronal circuits would therefore be maximally distinct. If the entirely different word forms “wish” and “desire” are being connected with the concepts, the resultant enlarged lexico-conceptual circuits would exhibit a reduced degree of overlap. The middle panel in Fig. 6 illustrates this mechanism. By cross-system association of word forms and concepts, and development of a higher-order semantic circuit, the degree of overlap between the widely distributed DNA/TCs is reduced (from 66 to 33 % in the example).

We submit that shrinking the overlap of cell assemblies by adding distinct assembly parts is the key evolutionary advance of the human brain. It may be the learned links between concepts and words that enabled humans to make so many and so fine-grained conceptual distinctions. The mechanism works both ways: also similar word forms, such as the words “pick” and “kick”, whose cell assemblies may overlap to a great extent and may be more easily separated functionally if they are linked to entirely different concepts (see bottom panel of Fig. 6). The mechanism is not limited to classic conceptual-linguistic discrimination. The huge human motor and action repertoire may be causally related to the possibility to verbally distinguish numerous minimally different action schemas. Even the abilities to perceptually distinguish colors, spatial locations, tastes, emotions, etc. may be driven, in part, by available linguistic tools. The overlap reduction by cross-modality binding of information may account for several of the features of specifically human cognitive processing discussed previously.

The suggestions that language drives cognition and perception had previously been made by scholars that viewed language structure as a driving force of cognitive and perceptual processes (von Humboldt 1836; Whorf 1956). Recent evidence has, in part, supported a role of language in spatial cognition, color perception and emotion processing (Regier and Kay 2009; Majid et al. 2004; Gendron et al. 2012; Winawer et al. 2007; Thierry et al. 2009). The cell assembly model now provides a tentative explanation *why* this may be so. As mentioned above, the overlap reduction idea also implies the reverse mechanism, the easier discrimination of linguistic tools, for example speech sounds, when they are used to make meaningful distinctions. Note that it is well established that speech sounds regularly used to distinguish concepts (for example the vowels [i] and [y] in French) are themselves easier to distinguish, whereas sound distinctions that do not serve a linguistic role in one’s language may not be perceived as well and elicit reduced acoustic-phonological brain responses (Näätänen et al. 1997; Diaz et al. 2008).

If shrinking cell assembly overlap by cross-modality binding of information is critical for human cognition, a range of further predictions results. Are there more powerful connections between the relevant brain systems (including perisylvian, visual and motor areas) in humans compared with their monkey relatives (for recent positive evidence, see Rilling 2014)? Would learning a new color term changes both behavioral color discrimination and the color-elicited brain response? Is the learning of a new action repertoire facilitated by concordant linguistic discrimination? Would lesions in the connection pathways between language, action and perception systems reduce the ability to perform minimally different actions and to make perceptual distinctions? And would it likewise impair the discrimination of words? Further questions apply in the translational domain: Would the teaching and therapy of language, action and perception in healthy people and neurologically impaired individuals profit from taking advantage of cross-modality information linkage? For example, one suggestion has been that aphasia therapy works more efficiently if the method applied takes into account the functional connections between language and action systems documented by neuroscience research (Berthier and Pulvermüller 2011). Finally, would neurocomputational networks that intrinsically interlink language, perception and action information through cross-modality links allow for more efficient modeling of cognitive functions than conventional models separating these systems functionally? Could these neurocomputational systems advance robotics in leading to more human like artifacts? A wealth of new research streams and translational perspectives is opened by the concept of distributed neuronal assemblies functioning as thought circuits, whose main advantage is to provide a mechanism for conceptual differentiation by overlap reduction and multimodal binding.

## Summary

Using Braitenberg’s brain-theoretical considerations about cell assemblies and their embedding into cortical neuroanatomy and function as a starting point (Braitenberg 1978), we explain how the concept of a widely distributed neuronal assembly, DNA, or thought circuit, TC, can model a range of cognitive processes. Crucially, this cell assembly perspective offers an explanation why cognitive brain processes are related to specific brain areas. Corticocortical connection structure and the loci of correlated neuronal activity during learning together explain the location of the areas relevant for language and visuomotor processing. The facts that higher cognitive processes of memorizing actions or taking decisions are not attributable to motor or sensory brain systems, but rather draw heavily on adjacent multimodal cortex, are explained by corticocortical connection structure, especially the high connectivity of these multimodal sites with sensorimotor areas and the consequent formation of DNA cores there. Category-specific meaning processes in brain areas specialized for particular semantic types are in part explained by correlation learning of word form and concordant modality-specific semantic information. Thus, instead of being restricted to a report of brain–cognition correlates, the concept of a widely distributed cell assembly interlinking information types, or DNA/TC, opens perspectives on explaining why mental processes map on their respective cortical areas. Over and above localization questions, the DNA/TC approach addresses functional questions, why attention to unknown materials is more important for their processing than for familiar signs such as a high-frequency word or one’s own name. DNAs also provide a mechanism for combinatorial generalization and for semantically linking words and concepts (or thoughts). Spelled out in terms of DNA/TCs, what makes humans special is the ability to interlink information between distant brain areas and thereby shrink the overlap between representations. Overlap reductions may be a key prerequisite for the emergence of huge vocabularies, action repertoires and conceptual systems.

## Footnotes

1 It is evident that the localization of cortical motor and sensory systems is driven by efferent and afferent neuroanatomical connectivity with effector and receptor organs. For the higher cognitive functions including working memory, decision, attention, language and conceptual thought, corresponding explanations are missing.

2 The authors indeed write: “ ...in order to keep an already complex computational framework manageable, we implemented the vATL semantic system alone. Specifically, it was *set to generate semantic outputs* for comprehension and *provided the semantic input* for speaking/naming” (p. 393, italics added by FP, MG & TW).

3 McClelland et al. (1986) proposed an analogy between cognitive theories and Newtonian vs. quantum field theory, making the point that the latter may lead to more precise explanations. We believe that their point does not apply to large parts of cognitive neuroscience, which, in our view, are still at a preexplanatory, descriptive stage when it comes to relating cognitive processes to brain function and structure. However, we acknowledge that neural network models have helped to better understand the relationship between correlational information and cognitive processing, which indeed implies a level of explanation.

4 Some of the “predictions” are already known to be true or false. Please note that the aim of the neurocomputational investigations summarized is not to recapitulate any active areas or ignition times, but rather to provide an explanatory pathway from established neuroscience knowledge and principles to the known facts—along with those to be revealed by future experiments.

5 This is reminiscent of Marr’s triune level theory, with representational-and-algorithmic between functional and microstructural levels (Marr 1982). However, the cell assembly approach goes further than that because it *unites* the three levels with each other (in the sense of Bechtel 1994; Darden and Maull 1977): a cell assembly may be seen as a correlate of a word or syntactic rule (functional level); it consists of neurons (micro-structure) and can be described mathematically (algorithmically) in terms of connection probability and structure, learning principles, functional dynamics and so on (Wennekers et al. 2006; Gerstein et al. 1989; Harris 2005).

6 In neurocomputational and experimental work, it may be useful to use a more precise mathematical definition (see, for example, Garagnani et al. 2009b).

7 For a discussion of mathematically precise formulations of relevant learning rules, see Garagnani et al. 2009b.

8 Please note that, although syllable articulation misses any visual aspects, correlations between speech and visual information about mouth movements can be mapped when observing others.

9 For an overview of related neurocomputational work on cell assemblies, see, for example, (Huyck and Passmore 2013; Wennekers et al. 2006; Palm 1982).

10 Neurons strongly linked into the assembly can also form outside areas strongly connected with each other, but this is less likely. So the lion’s share of the core part of cell assemblies emerges within the strongly interlinked multimodal areas.

11 A recent network model simulated a range of temporal features of activation dynamics in an associative circuit model of different cortical areas (Verduzco-Flores et al. 2009). However, this work did not aim at explaining why specific parts of the architecture showed memory activity in the first place.

12 There could be strong direct links between all word representations but the anatomy of the cortex speaks against this possibility. Assuming probabilistic connectivity, one word-related DNA of 1,000 neurons (see, for example, Palm 1993), with 10,000 synapses per neuron, could reach a maximum of $10^{7}$ other neurons. The human cortex includes about $1.5*10^{10}$ neurons (Haug 1987), ca. 1/10 of which are in the perisylvian language cortex (i.e., some $1.5*10^{9}$ neurons overall). As a maximum of $10^{7}$ neurons can be reached by way of a monosynaptic link from one DNA, a second DNA of 1,000 neurons in perisylvian cortex would only receive ca. 7 inputs (more likely even less, as some of the projections reach extrasylvian neurons) in the average, which is probably not enough to bring about a lasting functional change or ignition in the second DNA. With two synaptic steps, most (or even all) of the neurons in perisylvian cortex can be reached, and after strengthening of connections, these can become effective. This argument provides a rational for the assumption thatgrammatical sequence detection is based on indirect links between word-related cell assemblies (for discussion of this neuroanatomical argument, see Pulvermüller 2002; Pulvermüller and Knoblauch 2009).

13 This does not apply to all action words in everybody. Words related to actions not typically performed by humans (“barking”), to specialist actions (“pole jumping”) in nonspecialists and to standard human actions in persons with neurological motor deficits may primarily be learned in a perceptual manner or from linguistic context. It is an exciting topic of current research to investigate differences in action-based and perceptually based semantics in such cases.

14 An approach to category specificity has been offered in terms of conceptual mechanisms determined by the genetic code (Shapiro and Caramazza 2003). This perspective can explain inborn category distinctions that provide a selection advantage in natural environments, but fails to account for intercultural differences in category systems and does not explain the placement of cortical loci each specific category draws upon.

15 It has been argued that not only concrete concepts but abstract ones too are “grounded” in perception and action. This means that there is semantic knowledge about specific situations, action schemas and their context and so forth, to which the concepts apply (Barsalou and Wiemer-Hastings 2005). Consistent with this notion, neuroscience research showed that the processing of abstract emotion-loaded concepts such as WISH and DESIRE relates to brain activity in a range of brain structures with known roles in action and emotion processing (Moseley et al. 2012; Vigliocco et al. 2013). The mechanism of overlap reduction, which we outline below, is assumed to apply across cortical areas, including sensorimotor and multimodal cortices, and the limbic system.
